# 3D hydrogel models of the neurovascular unit to investigate blood–brain barrier dysfunction

**DOI:** 10.1042/NS20210027

**Published:** 2021-11-09

**Authors:** Geoffrey Potjewyd, Katherine A.B. Kellett, Nigel M. Hooper

**Affiliations:** 1 Division of Neuroscience and Experimental Psychology, School of Biological Sciences, Faculty of Biology, Medicine and Health, University of Manchester, Manchester M13 9PT, U.K.; 2 Geoffrey Jefferson Brain Research Centre, Manchester Academic Health Science Centre, Northern Care Alliance and University of Manchester, Manchester, U.K.

**Keywords:** blood brain barrier, extracellular matrix, induced pluripotent stem cells, neurodegeneration, stroke

## Abstract

The neurovascular unit (NVU), consisting of neurons, glial cells, vascular cells (endothelial cells, pericytes and vascular smooth muscle cells (VSMCs)) together with the surrounding extracellular matrix (ECM), is an important interface between the peripheral blood and the brain parenchyma. Disruption of the NVU impacts on blood–brain barrier (BBB) regulation and underlies the development and pathology of multiple neurological disorders, including stroke and Alzheimer’s disease (AD). The ability to differentiate induced pluripotent stem cells (iPSCs) into the different cell types of the NVU and incorporate them into physical models provides a reverse engineering approach to generate human NVU models to study BBB function. To recapitulate the *in vivo* situation such NVU models must also incorporate the ECM to provide a 3D environment with appropriate mechanical and biochemical cues for the cells of the NVU. In this review, we provide an overview of the cells of the NVU and the surrounding ECM, before discussing the characteristics (stiffness, functionality and porosity) required of hydrogels to mimic the ECM when incorporated into *in vitro* NVU models. We summarise the approaches available to measure BBB functionality and present the techniques in use to develop robust and translatable models of the NVU, including transwell models, hydrogel models, 3D-bioprinting, microfluidic models and organoids. The incorporation of iPSCs either without or with disease-specific genetic mutations into these NVU models provides a platform in which to study normal and disease mechanisms, test BBB permeability to drugs, screen for new therapeutic targets and drugs or to design cell-based therapies.

## Introduction

**Figure 1:**
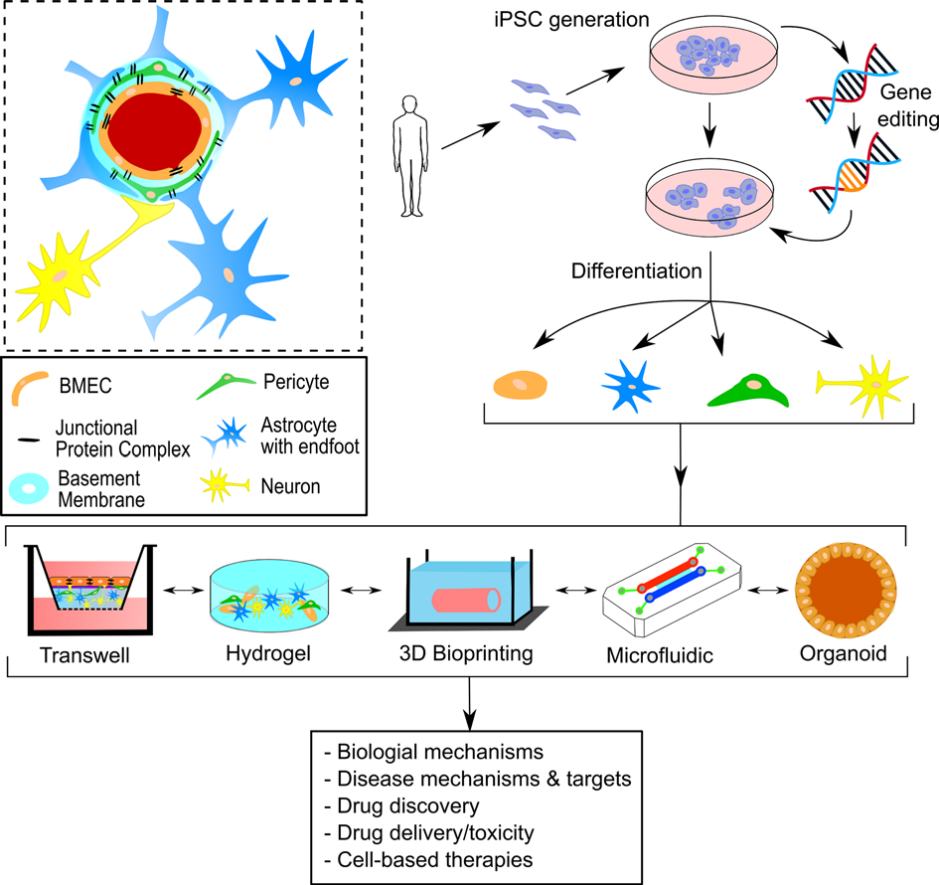
Using human induced pluripotent stem cells and biomaterials to generate 3D models of the NVU Human iPSCs either with or without gene-editing can be differentiated into the various cell types of the NVU. The cells, together with appropriate biomaterials that mimic the ECM, can be incorporated into a range of models of the NVU in which to study BBB function. Inset: schematic of the NVU. See text for details. Abbreviation: iPSC, induced pluripotent stem cell.

The neurovascular unit (NVU) combines the neural and vascular components of the brain in an important interface that maintains healthy brain physiology. The neural component of the NVU consists of neurons and glial cells (microglia, astrocytes, oligodendrocytes), and the vascular component consists of brain microvascular endothelial cells (BMECs), pericytes and vascular smooth muscle cells (VSMCs) (Figure 1). Together, the neural and vascular components of the NVU form the blood–brain barrier (BBB) that restricts permeability between the central nervous system (CNS) and peripheral tissues. In addition to the cells of the NVU, there is the non-cellular extracellular matrix (ECM) that provides structural support and biochemical cues for the NVU cells, allowing for cell adhesion and mechanical feedback between the cells and the extracellular environment. A specialised component of the NVU ECM is the basement membrane, which surrounds the BBB endothelium, encapsulates pericytes and provides different structural properties as compared with the surrounding parenchyma ECM. The vasculature in the brain is critical to providing oxygenated blood and nutrients to all parts of the brain, as well as removing waste products. Maintaining efficient cerebral blood flow (CBF), the integrity of the BBB and correct NVU function are imperative to sustain healthy brain function.

Neurovascular dysfunction and BBB breakdown have been observed in, and may contribute to the pathology of, a number of CNS disorders such as ischaemic acute stroke, small vessel disease, intracerebral haemorrhage, Alzheimer’s disease (AD), multiple sclerosis, amyotrophic lateral sclerosis, Lewy body diseases, traumatic brain injury/chronic traumatic encephalopathy and epilepsy [1–9]. NVU dysfunction can be a consequence of ageing and/or a variety of vascular risk factors that range from lifestyle habits to disease, such as smoking, diabetes, stroke and hypertension [10], with vascular oxidative stress and inflammation that arise from these risk factors causing the damage that results in NVU dysfunction.

To investigate the role of the NVU and BBB in health and disease, effective NVU models are required. Transgenic and surgically induced animal models of neurodegenerative diseases and stroke have provided important information on the function and dysfunction of the NVU. However, such animal models may not fully reproduce the human disease, thus limiting their effectiveness, as well as the limitations of translating findings from non-human models to the human situation [11]. *In vitro* models of the NVU provide an alternative accessible experimental platform which, when combined with NVU cell types differentiated from human induced pluripotent stem cells (iPSCs), can produce valuable models in which to study NVU structure and BBB (dys)function (Figure 1). As iPSCs can be differentiated into all of the major cell types of the NVU, there is the potential to build complex human NVU cell models (Figure 1).

The impact of disease-associated mutations on NVU structure and BBB function can be investigated in one of two ways. First, generating iPSCs from individuals with disease-associated genetic mutations and comparing the resulting differentiated cell properties with those obtained from ‘control’ individuals without the disease-associated mutation. However, due to the inherent genetic differences between any two individuals, multiple disease and control lines need to be studied to be certain that it is the disease mutation and not other genetic differences that are causing the observed phenotype. Alternatively, genetic editing of iPSCs through CRISPR-Cas9, for example, enables the introduction of a disease-associated mutation into a cell line (or correction of a mutation to the wildtype sequence) such that the only difference between the ‘disease’ and ‘control’ lines is the mutation of interest, so-called isogenic human disease models. Multiple iPSCs, including many isogenic lines, are available from repositories such as the European Bank for induced pluripotent Stem Cells (EBiSC; https://ebisc.org/), the New York Stem Cell Foundation (NYSCF; https://nyscf.org/) and the recently announced iPSC Neurodegenerative Disease Initiative (iNDI) [12].

However, key to a successful *in vitro* NVU model is the relative positioning of the different cell types within a 3D structure that mimics the mechanical and biochemical properties of the brain ECM and allows a functional BBB to form. In this review, an overview of the different cell types of the NVU and the structure and properties of the brain ECM is provided. The properties of various hydrogels, 3D porous biomaterials, which can be used to model the mechanical and biochemical properties of the brain ECM are discussed. In combination with iPSC-derived NVU cells, how these biomaterials can be used to create complex, robust and translatable *in vitro* models of the human NVU in which to study BBB function and dysfunction in disease is described.

## The components of the NVU

### Cellular components

Investigation of the NVU *in vitro* requires the culture of the different cell types, for example, through the use of immortalised lines, which are often criticised as being non-physiological, or the use of primary cells, which have limitations in the number of times they can be passaged. The advent of iPSCs, including those with disease mutations, that can be differentiated to a range of cell types has revolutionised *in vitro* cell studies providing a route to more physiologically relevant models. Protocols have been developed for the differentiation of iPSCs into BMECs [13,14], pericyte-like cells [15,16], VSMCs [17,18], neurons [19], astrocytes [20,21], microglia-like cells [22,23] and oligodendrocytes [24,25]. However, while iPSC-derived cell types are considered to be more physiologically relevant for disease modelling *in vitro*, they are not without their limitations. Neuron [26,27] and astrocyte [28] maturity has been a cause of concern as, despite generating iPSCs from aged donors, the process has been found to ‘reset’ the cell profile causing a loss of the ageing profile and resulting in the production of immature cells [26] which lack markers of ageing [27]. This obviously limits the application of such models in the study of diseases of ageing. However, more recently such concerns have been addressed with direct reprogramming methods shown to retain markers of ageing in both astrocytes [29] and neurons [30,31]. In addition, there have also been questions raised over the cell identity of BMECs [32] and the specific subtype of astrocytes [28].

**Figure 2:**
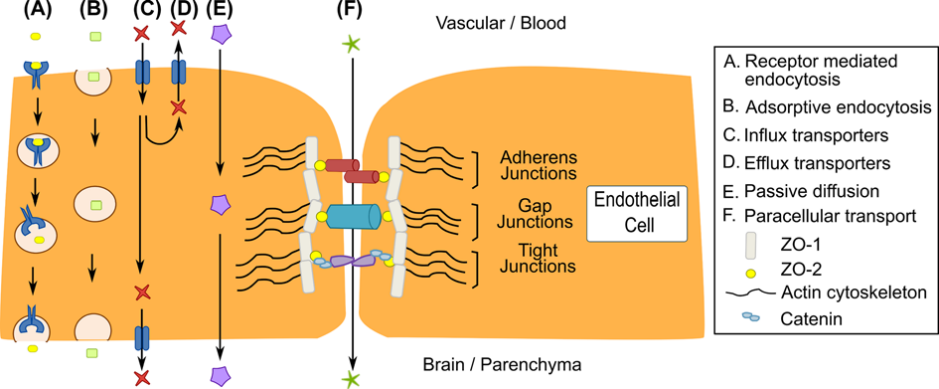
Transport mechanisms and junctional complexes of the BBB endothelium Various transport mechanism allow molecules to be moved across the BBB. (**A**) Receptor-mediated endocytosis involves the binding of a molecule to a receptor, triggering endocytosis and transport across the endothelium. (**B**) Adsorptive endocytosis is the transport across the endothelium by membrane encapsulation of a molecule. (**C**,**D**) There are multiple transporters expressed on the vascular and parenchymal membranes of the endothelial cells that facilitate the influx and efflux of molecules. (**E**) Small lipophilic compounds and molecules can passively diffuse across the membranes of the endothelial cell to access the CNS, although efflux transporters shown in mechanism (D) can remove these compounds back into the systemic circulation. (**F**) Paracellular transport between cells is limited due to the presence of junctional complexes between neighbouring cells. Scaffolding proteins consisting of complexes of ZO-1 and 2, along with catenin, link the ECM to the intracellular actin cytoskeleton through junctional complexes: adherens junctions, gap junctions and tight junctions.

BMECs are the primary cells of the vasculature of the BBB, forming a selectively permeable barrier, and developing a high level of functionality through interactions with other cells of the NVU and with the ECM. A key example of this is in neurovascular coupling, where the interaction between the capillaries and neuronal components regulates CBF [8,33]. In the BMECs various transport mechanisms, including influx and efflux transporters and receptor-mediated endocytosis, maintain the selective permeability of the BBB (Figure 2). Junctional complexes formed between neighbouring endothelial cells regulate paracellular permeability through the BBB, as well as transferring extracellular mechanical signals into the cell. There are three different classes of junctional complexes in the BBB endothelium: tight junctions, adherens junctions and gap junctions (Figure 2). In the junctional complexes zonula occludens (ZO) scaffolding proteins bind to the actin cytoskeleton and translate external mechanical stimuli into the endothelial cells [34]. BMECs differ from other endothelial cells in having specialised tight junctions, efflux transporter activity and reduced non-specific transcytosis [13].

Mural cells, namely pericytes and VSMCs, wrap around the endothelial cells of the BBB and have a fundamental role in vascular network formation [35]. These mural cells are encapsulated within the basement membrane, with VSMCs surrounding larger blood vessels and pericytes the smaller vessels such as arterioles, venules and capillaries, although there is not a clear cut divide, with pericytes gradually replacing the VSMCs as the vessels transition into capillaries [36]. Both pericytes and VSMCs have important roles in NVU functions, including regulation of BBB permeability through controlling junctional complex protein expression [37,38]. These vascular cells are also involved in the clearance of foreign molecules and toxins, in the regulation of CBF in arterioles and alterations of capillary diameter and tone, and in controlling neuroinflammation through reducing leucocyte trafficking in regions of blood vessels covered in pericytes [39–43].

Astrocytes are well-known for their roles in responding to injury within the CNS, recycling neurotransmitters, remodelling synapses, and aiding generation of new neurons [44,45]. Astrocytes are also crucial to the maintenance of a functional BBB, as revealed when tamoxifen-induced astrocyte ablation reduced ZO-1 expression in mouse brain blood vessels in areas without astrocytes, which was not compensated for by the presence of other NVU cells [46]. Astrocyte endfeet processes wrap around the basement membrane of approximately 99% of the endothelium, aiding development of junctional complexes [47]. Secreted glial factors are important mediators of endothelial barrier formation, while endothelial cells aid the development of astrocytes [48].

Neurons are important components of the NVU, with roles in neurovascular coupling, regulation of CBF, homoeostatic maintenance through regulating transporters in endothelial cells, and the formation and maintenance of vascular networks [49–53]. Neuronal cells are able to detect alterations in the supply of oxygen and nutrients to the brain tissue, and signal through astrocytes to the endothelium to increase CBF [54]. Secreted factors, such as vascular endothelial growth factor (VEGF) and brain-derived neurotrophic factor (BDNF), from both BMECs and neurons have been shown to be mutually beneficial towards formation and maintenance of the BBB and neural function in the NVU [55–57]. In the NVU, oligodendrocytes interact with endothelial cells to promote angiogenesis [58,59], and in turn, endothelial cells interact with oligodendrocytes to promote oligogenesis [59].

### The ECM

The ECM is a complex, well-organised non-cellular network of polysaccharides and proteins, which occupies the space between cells providing an essential structural support to the tissue. A relatively large proportion (17–20%) of the brain consists of ECM [60], which is key to the physical structure of the brain and has a dynamic composition dependent on the location and cell types present within that region [61]. The ECM is crucial to cell adhesion, migration, differentiation and mechanotransduction [60,62–64]. The brain ECM is primarily composed of glycosaminoglycans (e.g. hyaluronan), proteoglycans (e.g. neurocan, brevican, versican and aggrecan), glycoproteins (e.g. tenascin-R), and low levels of fibrous proteins (collagen, fibronectin and vitronectin) [65,66]. The low amount of collagen results in the low stiffness of the brain (see section ‘Characteristics of hydrogels to mimic the brain ECM’). Perineuronal nets are a CNS-specific ECM component that wrap around neurons in a lattice structure and have important roles in synaptic plasticity and stabilisation of synapses [67]. Perineuronal nets also promote angiogenesis and vascular integrity through release of growth factors and cytokines [68]. A thin fibrous component of ECM surrounds the brain endothelial vasculature, referred to as the basement membrane. The basement membrane is primarily composed of collagen type 4 (Col IV) and laminin, with fibronectin and proteoglycans integrated into the network [69]. Through mechanotransduction and biochemical cues, the basement membrane is critical to the development of junctional complexes within the BBB. From this brief overview, it is apparent that the multiple cell types within the brain, along with the surrounding ECM, work together to form a functional NVU.

## Dysfunction of the NVU

NVU dysfunction causes a reduction in CBF, disruption of the BBB, and a damaging immune and inflammatory response [70]. One of the hallmarks of NVU dysfunction is loss of BBB selective permeability with subsequent infiltration of various foreign proteins and cells into the brain parenchyma, including erythrocytes, leucocytes and antibodies. This in turn leads to induction of a pro-inflammatory phenotypic response from glial cells, with reactive astrocytes, microglia, and activation of pro-inflammatory macrophages causing further damage to the NVU [44,70–72]. Matrix metalloproteinases (MMPs) secreted from infiltrating leucocytes degrade junctional complexes and ECM components leading to degradation of the basement membrane, demyelination of neurons and lifting of pericytes and astrocytic endfeet from the vascular basement membrane [37,42,73–75].

Mutations in the protein components of the brain ECM are associated with neurovascular dysfunction and the onset of sporadic cerebral small vessel disease [76]. Global proteomic analysis revealed that genes related to cerebrovascular diseases, such as *COL4A1*, *COL4A2*, *VCAN* and *APOE*, were significantly enriched in the cerebrovascular ECM network [77] and increased expression of fibronectin, perlecan and Col IV was observed in the early stages of AD [78]. The basement membrane degrades and thins after neurovascular dysfunction (e.g. following stroke damage) and the expression of cellular ECM receptors, mainly integrins and dystroglycan, was reduced [79–81]. In diseases such as AD and stroke, this degradation of the ECM is mediated by MMPs [75]. In AD, MMP2 and MMP10 expression is higher, potentially leading to degradation of the tight junctions of the BBB and the breakdown of the basement membrane and brain parenchymal ECM [82]. In addition, a variety of MMPs (including NVU dysfunction-associated MMP9) have been shown to degrade structural elements of the ECM [83], and respond to changes in ECM stiffness that occur in ageing [84]. Following stroke there is also a loss of perineuronal net markers [85] and an infiltration of inflammatory cells, resulting from a breakdown in tight junctions and the degradation of the basement membrane, which in turn cause further changes to the ECM [75].

While NVU dysfunction is observed in a range of different conditions, the cause may be distinct in different diseases. Understanding cause-and-effect in terms of the onset and progression of NVU dysfunction is therefore key to delineating disease mechanisms and developing treatment strategies. For example, in ischaemic stroke and small vessel disease, endothelial cell dysfunction has been identified as key to the pathogenesis that occurs prior to BBB breakdown [86]. In neurodegenerative diseases, however, cerebrovascular abnormalities have been shown to arise as a result of abnormal protein processing. For example, in AD where tau [87] and amyloid-β [88], proteins that are the pathological hallmarks of the disease, have been shown to cause changes in CBF which affect neurovascular coupling, leading to deterioration of the BBB. How these effects are mediated is still under investigation, although amyloid-β has been shown to exert its effects on CBF by causing a disruption in pericyte contractility [89]. The studies highlighted here, along with many others, clearly show that the individual cell types of the NVU, as well as the surrounding ECM, are all critically involved in normal NVU and BBB function, and that changes to them contribute to the development of multiple neurological disorders. Critical to developing reliable models of the NVU is not only incorporating the individual cell types of the NVU but also recapitulating the essential properties of the surrounding ECM; in the next section the characteristics of biomaterials that can be used for this purpose are discussed.

## Characteristics of hydrogels to mimic the brain ECM

**Figure 3:**
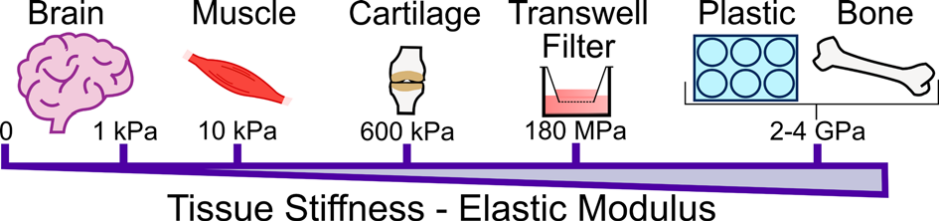
Stiffness in elastic modulus of different tissues and cell culture substrates The brain is one of the softest organs in the human body, with an elastic modulus at approx. 1 kPa. Muscle is some 10× stiffer, while cartilage is much stiffer at 600 kPa and the semipermeable membrane of a transwell filter has an elastic modulus of 180 MPa. Plastic and bone are extremely stiff substances with elastic moduli in the region of 2–4 GPa.

The brain is an extremely soft organ with a low shear modulus. Shear modulus is the quantified resistance to being deformed by a shear force and is used to classify the mechanical properties of biomaterials. Shear force can be measured using atomic force microscopy, but the most common method is oscillatory plate shear rheology where a force is applied to a biomaterial or tissue at different frequencies and levels of strain. This results in storage (G′) and loss (G′′) moduli, where the elastic component of the biomaterial is G′ and the viscous component is G′′. When referring to tissue stiffness this is related to the elastic component (G′) of the biomaterial or tissue. The low stiffness of brain is reflected by the low proportion of collagen type I (Col I) [90]; brain ECM stiffness has been recorded at 0.1–1 kPa, compared with muscle (another soft tissue) which has an ECM stiffness of 8–17 kPa (Figure 3) [91].

Usually when culturing cells, they are grown on plastic surfaces, such as flasks or plates, effectively in 2D [92]. Although growing cells in 2D on plastic substrates is relatively easy, providing for ease of manipulation and growth to large numbers, this approach does not take into account the 3D environment and mechanical properties of the native tissue in which the cells are present *in vivo*. In addition, plastics used in cell culture have a stiffness of approximately 1 GPa, significantly stiffer than brain tissue or the basement membrane (2–70 kPa) (Figure 3) [91,93,94]. Even the semipermeable membrane in transwell filters used in BBB models (see section ‘Development of NVU models’) is a much stiffer substrate than brain tissue, with an elastic modulus of 180 MPa (Figure 3) [95,96]. Clearly such plastic-based materials are not optimal materials to produce a physiologically mechanically relevant model of the brain.

The use of hydrogels allows cells to be grown in a 3D environment and with appropriate mechanical, biophysical and biochemical properties that better replicates the *in vivo* architecture of the native tissue [97]. Such hydrogels must be able to facilitate cell proliferation, allow for cells to produce and deposit native ECM, and provide suitable structural support for the cells. Hydrogels have the properties of a solid material but consist primarily of liquid. They are organised polymeric networks which allow for high absorption of water as well as the infiltration of cells and nutrients, preferably with low cytotoxicity, and the ability to be tailored to meet the mechanical and biochemical requirements of a specific cell type or as part of a tissue. Such mechanical and biochemical requirements include (i) tissue stiffness that mimics that of the native ECM, (ii) functional peptide groupings to bind to cell surface receptors and induce appropriate cellular phenotypic expression, and (iii) porosity that allows for the diffusion of gases, nutrients and waste products.

Hydrogels are typically stored in a liquid form before cross-linking and induction of gelation. This liquid to gel transition requires intermolecular organisation within the hydrogel by physical or chemical cross-linking, attaching polymer chains together and stabilising the polymeric chain in 3D. Chemical cross-linking involves covalent binding between polymeric chains to induce the liquid-to-gel transition [98–103], whereas physical cross-linking occurs through non-covalent interactions between polymer chains [104,105]. The mechanism of cross-linking itself can be toxic to cells. For example, certain chemical cross-linking techniques release cytotoxic reactive oxygen species during the redox reactions that are required to induce gelation [106], photopolymerisation through exposure to UV light may lead to cell damage [107,108], and the common chemical cross-linking agent glutaraldehyde is both mutagenic and neurotoxic [106,109]. It is important to consider the pros and cons with a particular hydrogel and the cross-linking method used. An advantage of physical cross-linking is that it avoids the potential cytotoxicity that can occur with chemical cross-linking, as well as having a very fast gelation time when the cross-linking process is ionic. However, a major disadvantage is that physical cross-linking gives less control than chemical cross-linking over porosity, cross-linking density and homogeneity [110,111]. A disadvantage of chemical cross-linking is that the resultant hydrogels typically exhibit an elastic behaviour which is detrimental to cell proliferation and migration [111], whereas physically cross-linked hydrogels have viscoelastic behaviour that better mimics native tissue microenvironments and enables more efficient cell differentiation, proliferation and spreading [111–113]. Light-based chemical cross-linking often allows the highest degree of resolution when bioprinting, but can also lead to cell damage when using UV-based light sources [107,108].

The presence of functional cell adhesion peptides (CAPs) within the polymeric network of a hydrogel enables cells to bind and interact with the hydrogel through cell membrane receptors, replicating the cell–matrix interactions found *in vivo*. When designing or selecting a hydrogel, it is important to consider the biological and functional properties of the CAPs present. Two of the most common amino acid sequences incorporated into hydrogels to provide cell adhesion are the tripeptide sequence Arg–Gly–Asp (RGD) and the pentapeptide sequence Ile–Lys–Val–Ala–Val (IKVAV). RGD domains within multiple different ECM proteins, including vitronectin and fibronectin [114], are the key binding sites for integrins and have been shown to facilitate neuronal and glial growth when present in an appropriate hydrogel [115]. IKVAV domains are present within laminin glycoproteins and have been shown to promote neurite outgrowth [116] and to improve tissue integration, as well as cell adhesion [117]. Aside from the two most common CAPs, RGD and IKVAV, there are a number of other CAPs which are less well-studied [118]. Given that different CAPs activate different cellular pathways following binding, the selection of a particular CAP for a hydrogel system can be intentionally used for either tissue specific or biological pathway specific activity. One example is the ‘QK’ peptide sequence Lys–Leu–Thr–Trp–Gln–Glu–Leu–Gln–Leu–Lys–Tyr–Lys–Gly–Ile (KLTWQELQLKYKGI), that mimics the receptor-binding domain of VEGF to induce angiogenesis and endothelial cell activation [119,120], and has been used in tissue engineering through covalent binding to an elastin-like polypeptide [98] and for the enhancement of endothelial cell activation following bone graft [120]. Similar hydrogel-based CAPs to the QK elastin-like polypeptide system could be incorporated into a hydrogel system, with the intention of inducing vascularisation or neurogenesis, or even to induce a disease phenotype like the accumulation of amyloid-β at the BBB. High-throughput screening techniques could also be employed to determine the effect of certain CAP, or CAP and hydrogel combinations, on cellular behaviour, enabling a more thorough investigation of a CAP which is appropriate for a specific model system. Protein-based polymers such as Col I have endogenous CAP domains for cell attachment, although other biopolymers, for example, alginate, gellan gum, chitosan and agarose, require chemical modification with a CAP domain to allow for cell adherence. There is also the possibility to mix non-functionalised polymers with naturally functionalised proteins to produce a hybrid hydrogel, benefitting from the structural stability of the polymer and the functionalisation of the native protein [121].

Depending on material type, concentration of biopolymer, and the cross-linking process that creates a hydrogel, the porosity of a hydrogel can vary dramatically, affecting the rate at which gases, nutrients and waste products diffuse through it [122]. The porosity of a hydrogel can be manipulated, for example, to promote vascularisation [123] and neural progenitor cell differentiation to neurons, oligodendrocytes and astrocytes [100]. Concentration gradients across hydrogels can potentially reduce the availability of essential nutrients and growth factors from reaching all cells within a 3D model [122,123]. Although concentration gradients can have a detrimental effect on cells in 3D culture, they may be critical to the formation of new tissues [124].

## Hydrogels relevant to NVU models

The neuronal and vascular components of the NVU have different ECM architecture and molecular composition. Thus, to create a physiologically relevant multicellular model of the NVU there is the need for hydrogels which mimic these different ECM characteristics. There are several biopolymers of either natural or synthetic origin that can be used as hydrogels for NVU models (Table 1). Natural biopolymers are extracted from living organisms and are further subdivided into protein-based and polysaccharide-based biopolymers. These are either constituents of the mammalian ECM or mimic the properties of the native ECM [125]. Synthetic biopolymers are non-natural, laboratory-synthesised biopolymers. Different biopolymers can be used to produce hydrogels that effectively mimic the brain ECM for the purpose of creating physiologically relevant NVU cell models.

**Table 1:** Hydrogels suitable for use in NVU models

Hydrogel biopolymer (source)	Gelation mechanism	Reported Elastic moduli (kPa)	CAPs	Biomaterial limitations	Advantages for NVU tissue engineering	References
Collagen type 1 (natural; protein)	Thermal; pH	∼0.1–3.3	Inherent	Limiting to angiogenesis; commonly combined with other material or cross-linkers to print	Cell-mediated remodelling through collagenases	[14,101,142,211–213]
Gelatin (natural; protein)	Thermal; photocross-linking	∼0.1–1	Inherent	Requires modification for 37°C cell culture; print close to gelation temperature	Cell-mediated remodelling through collagenases	[101,102,143,214]
Fibrin (natural; protein)	Fibrinogen cross-linking with thrombin	∼0.3–4	Inherent	Poor mechanical stability; combine with other materials to print	Widely used for neural tissue engineering; important role in angiogenesis	[142,165,215–219]
Alginate (natural; polysaccharide)	Ionotropic	∼0.2–3.5	Requires chemical modification or combining with other material	Unsuitable for BMEC culture; print with cross-linker; print into support matrix	Tuneable; NVU promoting cell adhesion domains chemically added	[141–143,220]
Gellan gum (natural; polysaccharide)	Thermal; ionotropic; photocross-linking	∼0.1–186	Requires chemical modification or combining with other material	Print with cross-linker; print into support matrix	Tuneable; NVU promoting cell adhesion domains chemically added	[102,221–224]
Hyaluronan (natural; polysaccharide)	Photocross-linking	∼0.01–3.5	Inherent	Risk of cellular damage from UV exposure	Promote cell migration, proliferation, angiogenesis and neurogenesis	[165,225–228]
Self-assembling peptide (SAP) (synthetic)	Self-assembly	∼1 to >50	Requires chemical modification or combining with other material	No inherent CAPs; expensive; depending on type form Aβ sheet structures	Tuneability; customisable bioprinting and cell adhesion domains	[104,163,229–234]
Elastin-like polypeptide (synthetic)	Self-assembly; photocross-linking; amine-reactive cross-linking	∼1 to >50	Requires chemical modification or combining with other material	If not self-assembling: require combination or printing with cross-linker	As with SAP; similar physical properties to vascular basement membrane	[98,164,233,235,236]
Poly ethylene glycol (synthetic)	Photocross-linking; thermal; ionotropic	∼0.9–132	Requires chemical modification or combining with other material	Chemical modifications; risk of cellular damage from UV exposure	As with SAP; well-documented usage	[237–241]

### Protein-based hydrogels

Collagen is a natural polymer and a key constituent of the ECM in many different tissues. There are several different types of collagen. Col I, the most abundant type of collagen, is typically found in tissues with a high elastic modulus, with Col IV being expressed in basement membranes and soft tissues like the brain. Even though Col I is primarily present in stiff tissues, it is a useful hydrogel for creating 3D models of the NVU as it can be tuned to the stiffness of the brain (G′ = 0.3–3.3 kPa) by altering either the concentration of Col I or the cross-linking mechanism [126]. In addition, it is an inexpensive alternative to Col IV. Col I has been used in multiple different studies to model the brain ECM and has been shown to be very effective in promoting the growth and attachment of NVU cells [121,122,127]. Col I also allows for cell attachment via integrin receptors expressed on the cells, thereby providing a mechano-transductive response from the ECM to the cell [121,128]. Col I can be dissolved in a dilute acid and neutralised with a base (allowing for storage in liquid form at refrigerated conditions), and then can be cross-linked simply by bringing the Col I solution to room or body temperature [129]. The mechanical stiffness of a collagen hydrogel can be altered by mixing with other hydrogel polymers, without compromising the cell adhesive properties of the collagen [121].

Matrigel is solubilised basement membrane ECM, extracted from Engelbreth–Holm–Swarm mouse sarcoma. Matrigel provides cells with a high concentration of laminins that are a major constituent of basement membranes, as well as proteoglycans, nidogens and Col IV [130,131]. Matrigel forms a loosely cross-linked gel that is used to coat plates for multiple different assays and cell growth protocols, including tube assays to assess angiogenesis in endothelial cells [132], and stem cell growth and differentiation of both neuronal cells and BMECs [19,133]. Plastic coated with Matrigel provides a basement membrane substrate for cells to attach to in an environment that is less stiff than untreated plastic. However, such models have limitations as the cell growth is still in 2D and 3D morphology is not developed to the levels attained when cells are encapsulated within a hydrogel. Other disadvantages of Matrigel include its diverse composition, the high variability between different batches reducing reproducibility of experiments, and its tumour-derived origin [134]. Matrigel is not included in Table 1 as it does not form a thick enough 3D network that can be measured by oscillatory plate shear rheology, and therefore the bulk mechanical forces that cells encounter when encapsulated in or on Matrigel are contributed by the surrounding culture materials (e.g. plastic) or the thicker hydrogel on which the Matrigel is coated [135].

Specific elements of the basement membrane can be used to provide the necessary cues for BMEC culture and functional BBB development, with the most common being a mix of Col IV and fibronectin [13,136]. A recent study established that Col IV-fibronectin is a suitable substrate for BMEC monolayer formation on top of soft Col I hydrogels, and also identified other basement membrane proteins, like agrin and perlecan, as appropriate basement membrane coatings [14].

### Polysaccharide-based hydrogels

Polysaccharides, such as chondroitin sulphate and heparan sulphate, are major components of the native ECM of both neural and vascular tissue [137,138]. Various polysaccharides can be used to create hydrogels that mimic the native ECM. For example, alginate, a polysaccharide similar to hyaluronic acid that is extracted from brown seaweed, has been used for many tissue engineering and regenerative medicine applications due to its biocompatibility with cells and tissues, and its fast cross-linking that occurs under physiological conditions which maintains cell viability during gelation [139,140]. As alginate does not contain inherent CAPs, it must be either covalently modified with functional CAPs [141] or blended with another hydrogel that does contain the necessary CAPs (e.g. Col I) [121] to enable cell adhesion. While alginate hydrogel has been used to enhance neuronal cell maturation in 3D culture [121], it does not enable adhesion or support proliferation of either peripheral endothelial cells [142] or BMECs [143]. Another natural polysaccharide is gellan gum which can form hydrogels that replicate the stiffness of *in vivo* brain tissue, although the hydrogels require the addition of cell binding motifs. Laminin can be added to the gellan gum to provide cell adhesion, thereby increasing the functionality of the hydrogel as demonstrated by neurons producing longer neurites [103].

### Synthetic-based hydrogels

Synthetic gels, such as polyethylene glycol, polyvinyl alcohol, poly(N-isopropylacrylamide), poly(2-hydroxyethyl methacrylate) and self-assembling peptide hydrogels, enable full control over the production of a hydrogel by allowing the user to decide the exact structural composition and functionalities depending on the tissue to be modelled [144]. Certain peptides have the physical properties that allow for self-assembly into β-sheets which form ordered nanofibrous hydrogel constructs that are ideal for tissue engineering projects [145]. To allow for cell adhesion, self-assembling peptides need to be chemically modified to contain cell-binding domains, such as IKVAV and RGD. These modifications can affect the structural and mechanical properties of the hydrogel, and such synthetic-based hydrogels can be significantly more expensive and less stable over prolonged culture times than hydrogels based on natural products.

## Outcome measurements of BBB functionality in NVU models

**Figure 4:**
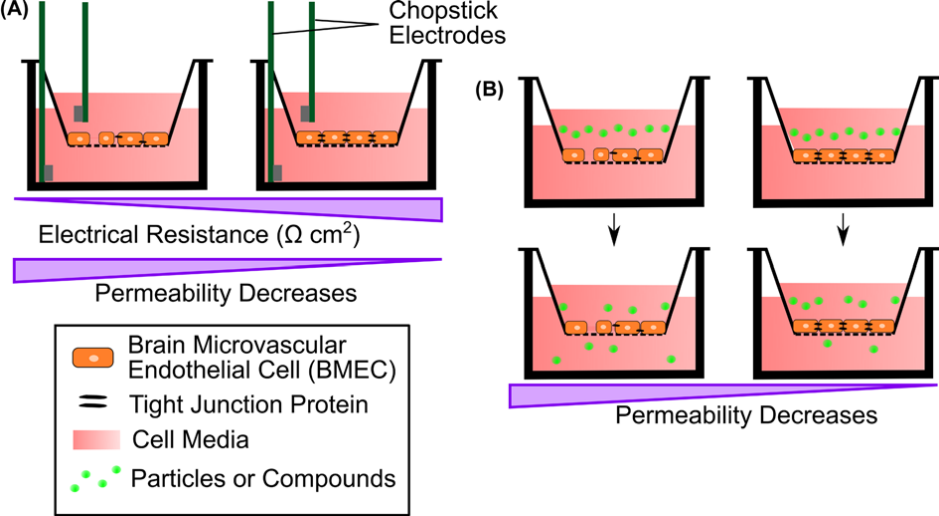
Techniques to measure BBB permeability in transwell models The transwell insert sits in the well and has a semipermeable base that allows passage of liquid between the upper and lower reservoirs. (**A**) TEER measurements are taken across the endothelial cell monolayer using ‘chop stick’ electrodes. Electrical resistance increases when the BBB is more fully formed equating to a lower permeability. Tissue resistance is measured in Ω, and the normalised UAR of the BBB is measured in Ω cm^2^. (**B**) Permeability to compounds of known size can be used to assess the permeability of the BBB. Following the addition of a fluorescent or other tagged molecule to the upper chamber, the amount of the molecule appearing in the bottom chamber can be measured over time, enabling a calculation of permeability across the BBB to be calculated.

When developing NVU models, a key consideration is the ability to measure cell function and BBB integrity. The relative permeability of the BBB can be measured in several ways. A common approach is to measure the electrical resistance across the BBB with a technique called transendothelial electrical resistance (TEER), which uses electrical resistance between two electrodes placed across the BBB as a surrogate measure of permeability (Figure 4A). TEER measurements are often used in combination with growing endothelial cells in a transwell model (see section ‘Development of NVU models’). The higher the resistance, the lower the permeability of the BBB. TEER values can be normalised to unit area resistance (UAR; Ω cm^2^) which is dependent on the surface area of the substrate that the endothelial monolayer is cultured on. TEER measurements can be used to compare the phenotype of *in vitro* BBB models to *in vivo* BBB measurements, where in rats and frogs UAR values from TEER recordings are in the range of 1200–1900 Ω cm^2^ [146–148]. BBB models incorporating human iPSC-derived BMECS can produce extremely high TEER values; for example, UARs between 1000 and 3000 Ω cm^2^ have been recorded [146–152]. While TEER is a useful measure of BBB integrity *in vitro*, it does not factor in any alterations that may occur to specific transporters at the BBB, and to investigate this, other more specific measurements are required.

The permeability of the BBB can be assessed by measuring the transport of compounds of known molecular weight across the BBB (Figure 4B) [146,153,154]. This can either examine non-specific transport, or specific transport through transport proteins. For non-specific transport, typically a fluorescent-based compound (e.g. Lucifer Yellow) or a compound with a suitable tag that is detected by a fluorescence-based assay (e.g. Rhodamine dextran) are used to determine the concentration of compound that has crossed the BBB [155]. For specific transport processes, compounds which only cross the BBB through transporter proteins at the endothelial cell surface are used. An example of this is glucose which is transported across the BBB by solute carrier transporters. By measuring the concentration of glucose either side of the BBB, the rate of transport through its specific transporters can be obtained. These techniques are commonly used with transwell insert models but can also be used in hydrogel and microfluidic models.

The interaction of endothelial cells through junctional complexes at the BBB is crucial to controlling permeability and overall BBB functionality (Figure 2) [72,156]. Additionally, the interactions between endothelial cells and other cell types within the NVU influence both BBB development and maintenance. The level of expression of junctional complex proteins, as well as their localisation within the cell, change as the endothelial layer matures and are influenced by interactions with other cell types, and can be readily measured by immunohistochemistry. Common proteins to investigate in junctional complexes are those that join adjacent endothelial cells, such as claudin-5 and occludin, to maintain a low paracellular permeability and high TEER [157–159]. An important protein for maintaining BBB integrity is N-cadherin which is involved in adherens junctions between endothelial cells and pericytes, and Connexin 43 in gap junctions (Figure 2).

While standard immunohistochemistry techniques and protocols can be used to stain cell-laden hydrogels, the incubation periods, concentration of antibody used, and the level of mechanical agitation may need to be increased to ensure that there is complete distribution of reagents throughout the hydrogel. Hydrogels can be optically transparent, but often become turbid when cross-linked (e.g. PureCol) making imaging difficult with large thickness gels. As hydrogels are heavily water-based (∼90%) tissue histology techniques, such as paraffin embedding, that involve dehydration and heating, deform the hydrogels and thus do not preserve the cell–cell and cell–matrix interactions that have formed in culture [160]. It is possible with a series of treatments in the freezing process to cryosection hydrogels [161]. An alternative technique that does not require the potentially damaging steps of paraffin embedding and cryosectioning, is that of agarose embedding and subsequent tissue sectioning using a vibratome [162], which allows for enhanced penetration of antibodies into the hydrogel slices and increased resolution of the resulting immunohistochemistry images. The spatial localisation and expression level of key structural proteins, transporters and cell–matrix adhesion proteins can be identified and quantified using a variety of techniques, including immunohistochemistry, enzyme-linked immunosorbent assay (ELISA) and Western blotting [163–165].

Changes which occur at the transcript level can also be utilised as an outcome measure in *in vitro* BBB models. However, an issue with using reverse transcription quantitative PCR (RT-qPCR) in a multicellular BBB model is mapping transcript changes to a single cell type, as the mRNA from other NVU cells will interfere in the measurements. One way to avoid this is to use cells from different species, so that species-specific primers can be used for identifying transcript changes. Alternatively, cells can be extracted from the hydrogel and separated by flow cytometry, although enzymes used to digest the polymer network may degrade the cellular material to be measured or interfere in the subsequent detection [166]. Changes in junctional complex mRNA expression in brain endothelial cells generally correlate with the protein level changes and functional changes in TEER [167]. However, in iPSC-derived BMEC junctional complexes, there was a disconnect between mRNA and protein level expression, with only protein level alterations directly affecting BBB integrity measured through TEER [168]. This highlights that functional permeability measurements, such as TEER, may be better correlated with protein, rather than mRNA transcript, expression in certain NVU *in vitro* models. More sophisticated approaches, such as single-cell RNA sequencing, transcriptomics and proteomics, can be used to determine the expression levels and profiles of multiple targets in complex NVU models.

## Development of NVU models

The NVU is a complex structure with multiple components, and various cell–cell and cell–matrix interactions. In this section, the techniques in use to incorporate multiple different cell types with appropriate ECM in order to develop robust and translatable NVU models are presented.

### Transwell models

**Figure 5:**
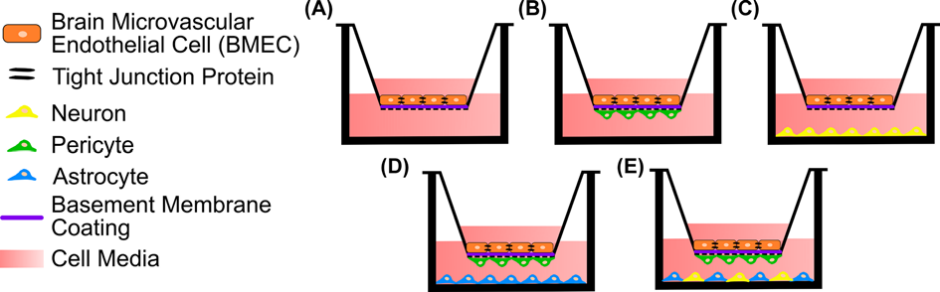
Transwell insert models of the NVU (**A**) Monoculture: BMEC monolayer on the surface of the insert without any other cell types. (**B**) Co-culture: pericytes, or any other adherent cells, are seeded on the underside of the transwell insert and BMECs are seeded on the upper surface. (**C**) Co-culture: cells, e.g. neurons, are seeded on the base of the well underneath the transwell insert, with BMECs on the insert. (**D**) Tri-culture: one cell type is seeded on the underside of the transwell insert, another cell type to the base of the well underneath the insert, along with BMECs on the upper surface of the insert. This allows for each cell type to be cultured on their own substrate with limited physical contact. (**E**) Quad-culture: pericytes are seeded on the underside of the transwell insert, with astrocytes and neurons co-cultured on the base of the well, with BMECs seeded on top of the transwell insert.

Transwell inserts are widely used for the development of BBB models as the semipermeable membrane of the insert provides a surface on which endothelial cells can be grown and measurements of permeability obtained with relative ease. When developing more complex NVU models, endothelial cells can be grown on the upper surface of the transwell insert to form a functional barrier (Figure 5A). Other cell types can be grown on the base of the well or the underside of the insert. These combinations provide three separate surfaces to culture cells on, with the possibility of co-culture (Figure 5B,C), tri-culture (Figure 5D) or quad-culture (Figure 5E). Co-culture in this model allows biochemical secretions from one cell type to diffuse through the media and affect the endothelial cells (and *vice versa*), however, there is a lack of direct physical contact between the cell types, although limited cell contact can occur through the pores of the insert, depending on the pore size, when cells are co-cultured on the upper and lower sides of the semipermeable membrane (Figure 5B). Appropriate membrane pore size can allow cell migration [169] and limited physical contact with the endothelial cells through cell processes from astrocytes [170–173], neurons [174] and pericytes [173]. A major limitation of such simple transwell models is that all the cell types are grown in 2D on stiff substrates that do not recapitulate the mechanical and biochemical properties of the native ECM.

### Hydrogel models

Hydrogels allow for the growth of cells in 3D and provide appropriate mechanical and biochemical cues for the cells. Hydrogels can be used to build models of the NVU either through encapsulating cells within the hydrogel or by using the hydrogel as a surface substrate on which to grow the cells. For example, iPSC-derived BMECs, pericytes and astrocytes have been encapsulated in Matrigel to provide a 3D ECM for self-assembly that recapitulated the anatomical and physiological properties of the human BBB *in vitro* [175].

**Figure 6:**
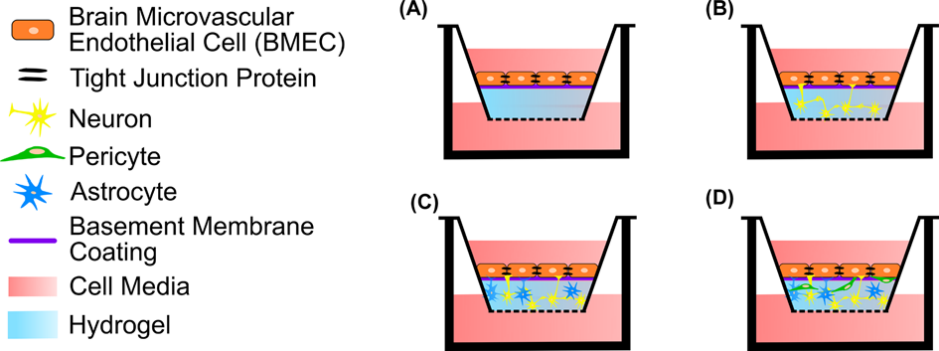
Transwell insert models of the NVU incorporating a hydrogel component Use of an NVU-mimetic hydrogel within the transwell insert allows the NVU cells to be grown in 3D beneath a BMEC monolayer without having separate compartments that physically separate the different cell types. (**A**) Monoculture: BMECs grown on a hydrogel that mimics the physical properties of the NVU ECM with a basement membrane coating to facilitate BMEC monolayer development. (**B**) Co-culture: BMECs are seeded on top of a basement membrane-coated hydrogel layer that is laden with another cell type, e.g. neurons, thereby enabling physical and biochemical interactions between the two cell types. (**C**) Tri-culture: neurons and astrocytes, for example, are both encapsulated within the hydrogel and cultured in 3D allowing both cell types to interact with each other within the hydrogel and with the BMEC monolayer. (**D**) Quad-culture: neurons, astrocytes and pericytes, for example, are grown within the 3D hydrogel layer with BMECs on the surface.

The lack of physical interaction between the endothelial cells and other cell types in the transwell models (Figure 5) can be overcome by introducing a hydrogel component on to the transwell insert (Figure 6). This allows for encapsulation of non-endothelial NVU cells in a 3D hydrogel matrix to replicate the brain parenchyma, rather than having a stiff planar seeding surface. A basement membrane coating can be applied to the surface of the hydrogel on which the BMECs can be grown (Figure 6A), to improve upon the non-physiological surface of the transwell insert. The complexity of the NVU can be built beneath the endothelial cell layer using other NVU cell types such as neurons, astrocytes or pericytes (Figure 6B), which all form physical interactions with the BMECs *in vivo*. By utilising the hydrogel as a brain parenchyma ECM there is the opportunity to culture multiple cell types within the hydrogel where they can form their own interactions and also interact with the BMECs, in tri-culture (Figure 6C) or quad-culture (Figure 6D) configurations, but still allowing for the use of permeability measurements such as TEER.

A vessel can be fabricated within a hydrogel by using a sacrificial element that the surrounding hydrogel can cross-link around and, when the sacrificial element is removed, leave an empty channel in the hydrogel that mimics the vessel. This can be achieved by simply embedding a glass rod or needle in the hydrogel during gelation that is removed afterwards [176]. The wall of the channel can then be coated with basement membrane proteins that enable BMEC attachment and the formation of a functional BBB [14]. More complex vessel structures can be modelled using 3D bioprinting and sacrificial biomaterials or microfluidic systems.

In addition to incorporating the multicellular nature of the NVU, the use of 3D hydrogels has significant advantages for the investigation of CNS disorders *in vitro.* A major limitation of growing cells in 2D in a plastic dish is that removal of the cell culture media during passaging and feeding results in the constant removal of the cellular secretome. This prevents cells from influencing their own extracellular environments and, in the case of many CNS disorders, preventing the build-up of extracellular proteins that may influence disease, such as amyloid-β in AD. The use of hydrogels has allowed the visualisation of amyloid-β deposits in cell culture, with these deposits giving rise to tau pathology that was reversed following administration of compounds that prevented amyloid-β production [177,178].

### 3D-bioprinting

The combination of a bioprintable hydrogel with cells to create a bioink enables the use of 3D-bioprinting to produce complex multicellular 3D models. For such an approach, the biomaterial to be used must be printable and the bioprinting process must not adversely damage the cells. The latter consideration is an issue as the best resolution 3D-bioprinting techniques utilise chemical cross-linking (UV; reactive oxygen species; glutaraldehyde) that is potentially toxic to cells [106–109,179].

**Figure 7:**
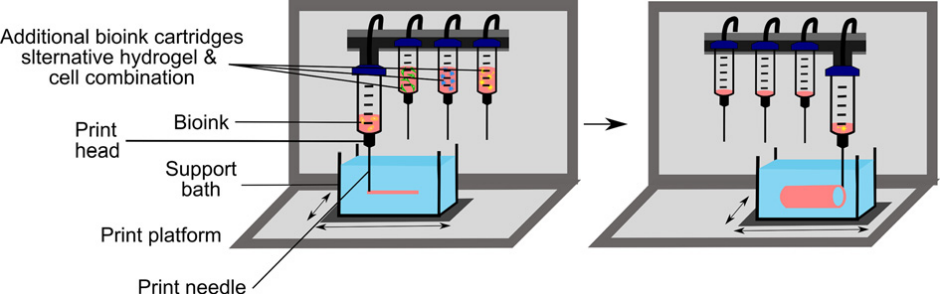
Schematic showing the use of bioprinting to manufacture an NVU model that represents a brain blood vessel Individual NVU cells are combined with a bioprintable pre-hydrogel solution to create a bioink that will replicate the microenvironment of the brain parenchyma. The bioinks are then placed into different printing cartridges and deposited using a computer-aided design template into a support bath that maintains the structural composition of the design. The support bath is filled with a specially formulated polysaccharide hydrogel that has liquid properties under shear force, allowing the bioink to be deposited in precise spatial locations. The pre-hydrogel solution is then cross-linked, and either removed from the support bath or kept in the support bath and placed within a 37°C incubator for tissue development and experimentation. See text for further details.

Spatial resolution is also an important consideration when bioprinting NVU models. The reported mean distance from a neuron to a capillary is ∼8 μm, and capillary to capillary is 40 μm [180], which is a spatial resolution that cannot be achieved currently by cell-friendly printing strategies [111]. Extrusion-based 3D-bioprinting which avoids exposure to damaging *in situ* chemical cross-linking agents results in high cell viability upon printing, and where resolutions of ∼20 µm have been achieved when using suspended layer additive manufacturing techniques [181,182]. Suspended layer additive manufacturing utilises the visco-elasticity of a self-healing fluid gel to support the structure of the hydrogel during deposition, avoiding gel flow and providing time for cross-linking to occur, after which the fluid gel can be removed, leaving a solid cross-linked 3D hydrogel construct (Figure 7). Other extrusion techniques, such as pneumatic and piston, suitable for bioprinting the NVU, have a maximum resolution of ∼200 µm.

Other techniques that can be used to avoid potentially harmful 3D-bioprinting, at the cost of precise spatiotemporal cell deposition, include manual casting of cell-laden hydrogels that allow cells to be dispersed throughout the hydrogel. This enables physical contact between cells encapsulated within the hydrogel, depending on porosity and cell seeding density, and physical cell–cell contact when endothelial cells are seeded on top of the manually cast hydrogel. To achieve higher resolution capable of replicating nanometre microstructure, a light-based 3D-bioprinting method is required, such as stereolithography or 2-photon-polymerisation. However, light-based chemical cross-linking may damage or alter the differentiation of the printed cells, and therefore such approaches are not currently suitable for NVU models. As photocuring chemicals and the technologies implemented become more advanced, there is the possibility of using non-damaging light sources for chemical cross-linking, thereby allowing fast cross-linking and high-resolution 3D tissue environments to be created, at least recreating the physical proportions of the larger blood vessels of the BBB [111].

### Microfluidic models

Like transwell models, microfluidic models can be used to culture cells in separate compartments and allow the integration of hydrogels to permit 3D cell culture, but importantly microfluidic models incorporate channels for the flow of media. A pump system can be used to circulate fluid through the microfluidic chip producing a shear force on the endothelial cells. Microfluidic chips are available commercially (e.g. AIM Biotech 3D Cell Culture Chip and Synvivo chip) or can be created through using additive manufacturing techniques. Such microfluidic chips have specific compartments in which the different parts of the NVU can be modelled. In some microfluidic models a BBB can be biofabricated by coating a chamber in basement membrane protein and seeding endothelial cells on the walls of the channel [183–186]. By using side channels separated by a hydrogel-filled central chamber, in conjunction with iPSC-derived BMECS, pericytes and astrocytes, it is possible to create a self-assembled microvascular network model within the microfluidic chip [187–190]. Such a model allowed capillaries with extremely low diameters to form and, when co-cultured with either pericytes or astrocytes, the diameters of the capillaries reduced further with the lowest and more defined diameter capillary and lowest permeability recorded when pericytes and astrocytes were co-cultured together with BMECs [188,189].

The pulsatile flow from blood in the circulatory system creates a shear force over endothelial cells which when applied *in vitro* increased the expression of junctional complexes (tight junctions and adherens junctions) and a range of multidrug resistance transporters in human primary brain endothelial cells [167]. Surprisingly, BMECs differentiated from iPSCs may not respond in the same way to shear stress as primary and immortalised cell lines. iPSC-derived BMECs did not alter conformation to align and elongate with the flow of shear stress, probably due to substantial and well-formed tight junctions developing under static conditions which decreased the motility and prevented morphological changes occurring in the BMECs upon addition of flow [168]. An advantage of a microfluidic chip is that the clear plastic that is commonly used and the small dimensions of the chip allow for microscopy and imaging, although this is dependent on the particular chip being used. A disadvantage with microfluidic chips is that the small dimensions of the chip mean that even when cells are grown in a hydrogel within the chip, due to the small volumes involved the major factor influencing cell mechanotransduction is the surrounding stiff plastic [135].

### Organoids

Organoids are ‘mini-organ-like’ tissues grown from stem cells into a 3D tissue-like structure. For neuroscience research, organoids are typically grown into a ‘mini-brain’ consisting of neurons that form a clump of cells, depositing their own ECM [191]. Cerebral and cortical brain organoid cultures will inherently have astrocytes, and astrocytes have been co-cultured with a brain organoid to make an ‘asteroid’ culture, which is a further step forward to studying cell–cell interactions using this model [192]. While organoid cultures are useful, they have limitations, such as variable organoid size and morphology obtained with different iPSC lines, limited oxygen and nutrient diffusion with consequent necrosis, limited maturation and the absence of some cell types found in the brain [193]. Attempts to address these limitations include using bioengineered fibre scaffolds to produce specific cellular configurations and facilitate formation of larger organoids [194] and using spinning bioreactors with smaller volumes of media and better controlled conditions [195]. A major issue in terms of developing organoid NVU models is the difficulty of incorporating vasculature within the cell mass [196]. The lack of a defined vasculature makes the measurement of permeability either exceedingly difficult or impossible. The thick mass of cells in an organoid also makes the visual assessment of tissue development difficult, with terminal measures of histology being needed to assess this. In addition, the thick mass of cells can induce a necrotic region in the centre from lack of nutrients, which may affect cell signalling and create biological variability within the tissue [197,198].

Recently though, attempts have been made to vascularise organoids by including endothelial cells in the cell mass, with expression of key junctional complex markers [199–201], a TEER measurement of 280 Ω cm^2^ [199], and tubular-like structures [199,201] being obtained, and the formation of perfused blood vessels when the vascularised organoids were implanted subcutaneously in mice [199]. In another example, an *in vivo* model of functional and vascularised human brain organoids was achieved by transplanting human brain organoids into the adult mouse brain [202]. Engineered organoid models which combine either hydrogel or microfluidic devices and organoid technology to improve the replicability, maturation and functional readouts achieved from organoids are being developed [203]. Vascularisation has also been achieved by using 3D-printing of a microfluidic chip to ensure precise spatial locations, and thereby interactions, between neural organoids and vascular tissue [204]. Combining a microfluidic device with a decellularised human brain tissue-derived ECM was used to recreate brain-mimetic niches necessary to guide neural and glial differentiation for brain organogenesis [205]. These examples clearly indicate that despite the current limitations of organoid models, by combining them with hydrogel, microfluidic and 3D-bioprinting technologies these models are clearly bringing us closer to being able to replicate the complexity of the NVU *in vitro*.

## Concluding remarks

Historically, investigation of neurological disease mechanisms and the development of treatment strategies both for acute and chronic conditions has been predominantly neurocentric. This has, however, begun to shift as the importance of non-neuronal components to disease mechanisms and pathology is more widely recognised, with the critical role of the NVU in maintaining homoeostasis in the brain gaining evidence. Given that many neurological disorders are specific to humans, animal models, even those incorporating human genes and disease-specific mutations, rarely recapitulate the human phenotype thus limiting their use in understanding disease mechanisms and in the translation of therapeutic targets to effective treatments.

Clearly, the development of robust, translatable models of the NVU has applications to the study of multiple neurological conditions, including emerging conditions such as Long Covid. SARS-CoV-2 infection has been shown to cause detrimental neurological effects in a number of Covid-19 patients [206] and the virus can infect both astrocytes and neurons [207,208]. Whether the virus itself is able to cross the BBB is still not clear, but the viral spike protein can infiltrate the brain via the BBB [209]. Further investigation, including using models of the NVU as described above, is clearly required to fully understand the neurological consequences of SARS-CoV-2 infection to provide understanding and enable treatment of the ‘brain-fog’ associated with Long Covid [206].

The advent of iPSC technology has revolutionised the study of the human brain and of neurological diseases, with the ability to create all the cell types of the NVU allowing the contribution of each cell type to normal biology and disease pathology to be investigated. To create relevant models of the NVU a key factor to incorporate is the ECM that provides mechanical support and biochemical cues to the cells of the NVU. Just as the native ECM acts as a scaffold for cells in the brain, hydrogel matrices act in the same way in 3D *in vitro* models, providing a platform for cell adherence and migration, and inducing mechanical cues. The combination of iPSC technology with reverse engineering approaches using hydrogels that mimic the properties of the native ECM, along with 3D-bioprinting, microfluidic devices and organoid development, has enabled various models of the NVU to be developed in which BBB functionality can be reliably assessed. These iPSC-based models of the brain and NVU are proving useful not only for disease modelling but also for drug development and screening [210]. The challenge for the future is how to further develop and/or combine these approaches to generate more robust models of the human NVU for the study of normal biology, to interrogate disease mechanisms, to assess the efficacy and safety of drugs, and as the basis for cell-based therapies.

## Data Availability

All supporting data are included within the main article.

## Abbreviations

AD: Alzheimer’s disease
BBB: blood–brain barrier
BMEC: brain microvascular endothelial cell
CAP: cell adhesion peptide
CBF: cerebral blood flow
CNS: central nervous system
Col: collagen
ECM: extracellular matrix
iPSC: induced pluripotent stem cell
MMP: matrix metalloproteinase
NVU: neurovascular unit
TEER: transendothelial electrical resistance
UAR: unit area resistance
VEGF: vascular endothelial growth factor
VSMC: vascular smooth muscle cell
ZO: zonula occludens
